# 2,9,10-Trimeth­oxy­dibenzo[*b*,*d*]oxepin-7(6*H*)-one

**DOI:** 10.1107/S1600536811053803

**Published:** 2011-12-21

**Authors:** Yan-Jun Hou, Shu-Lin Song, Wen-Yi Chu, Zhi-Zhong Sun

**Affiliations:** a College of Chemistry and Materials Science, Heilongjiang University, Harbin 150080, People’s Republic of China

## Abstract

The title compound, C_17_H_16_O_5_, was prepared through a cyclization reaction of 2-(3′,4′,5-trimeth­oxy­biphenyl-2-yl­oxy)acetyl chloride. The two benzene rings form a dihedral angle of 34.55 (5)°. The crystal structure does not feature any hydrogen bonds.

## Related literature

For general background to the synthesis and properties of the title compound, see: Suau *et al.* (1996 ▶); Tandon *et al.* (2009 ▶). For the biological activity of meth­oxy­dibenzooxepin-one derivatives, see: Yoshioka *et al.* (1978 ▶).
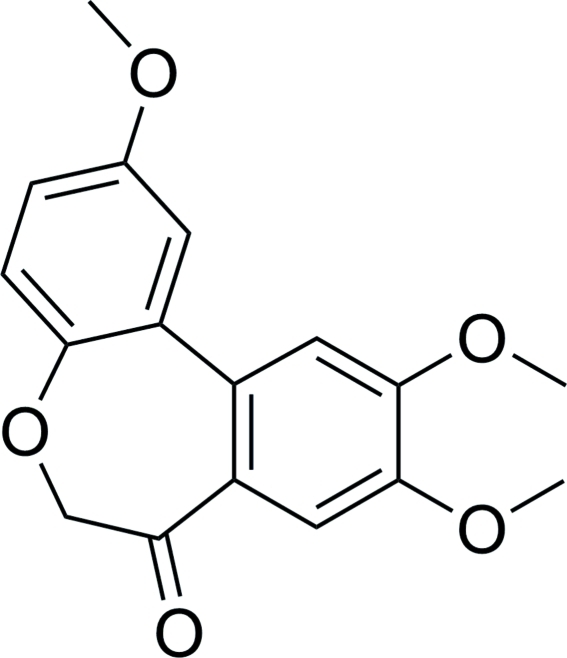


## Experimental

### 

#### Crystal data

C_17_H_16_O_5_
*M* *_r_* = 300.30
Monoclinic, 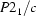

*a* = 11.5474 (10) Å
*b* = 8.3776 (7) Å
*c* = 14.8801 (13) Å
β = 93.607 (1)°
*V* = 1436.6 (2) Å^3^
*Z* = 4
Mo *K*α radiation
μ = 0.10 mm^−1^
*T* = 295 K
0.32 × 0.26 × 0.24 mm

#### Data collection

Bruker SMART APEXII CCD diffractometer
Absorption correction: multi-scan (*SADABS*; Sheldrick, 1996 ▶) *T*_min_ = 0.968, *T*_max_ = 0.976
11231 measured reflections
3517 independent reflections
2517 reflections with *I* > 2σ(*I*)
*R*_int_ = 0.022

#### Refinement

*R*[*F* ^2^ > 2σ(*F* ^2^)] = 0.044
*wR*(*F* ^2^) = 0.127
*S* = 1.05
3517 reflections
202 parameters
H-atom parameters constrained
Δρ_max_ = 0.26 e Å^−3^
Δρ_min_ = −0.18 e Å^−3^

### 

Data collection: *APEX2* (Bruker, 2004 ▶); cell refinement: *SAINT* (Bruker, 2004 ▶); data reduction: *SAINT*; program(s) used to solve structure: *SHELXS97* (Sheldrick, 2008 ▶); program(s) used to refine structure: *SHELXL97* (Sheldrick, 2008 ▶); molecular graphics: *SHELXTL* (Sheldrick, 2008 ▶); software used to prepare material for publication: *publCIF* (Westrip, 2010 ▶).

## Supplementary Material

Crystal structure: contains datablock(s) I, New_Global_Publ_Block. DOI: 10.1107/S1600536811053803/rk2322sup1.cif

Supplementary material file. DOI: 10.1107/S1600536811053803/rk2322Isup2.cdx

Structure factors: contains datablock(s) I. DOI: 10.1107/S1600536811053803/rk2322Isup3.hkl

Supplementary material file. DOI: 10.1107/S1600536811053803/rk2322Isup4.cml

Additional supplementary materials:  crystallographic information; 3D view; checkCIF report

## Figures and Tables

**Fig. 1.**
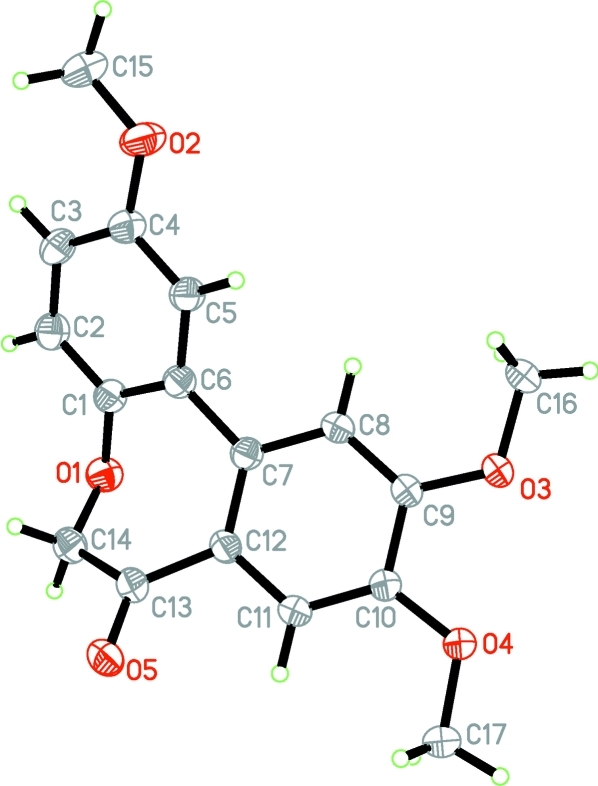
The molecular structure of the title compound with the atom numbering scheme. Displacement ellipsoids are drawn at the 50% probability level. H atoms are presented as a small spheres of arbitrary radius.

## supplementary crystallographic information

### Comment

The methoxydibenzooxepin–one derivatives have important antiviral activity and cause attention to new synthetic methods and investigation of similar compounds (Yoshioka *et al.*, 1978). Many methoxydibenzooxepin–one derivatives directly prepared from methoxy 2–(biphenyl–2–yloxy) acetyl chloride (Suau *et al.*, 1996; Tandon *et al.*, 2009). The 2,9,10–trimethoxydibenzo[*b,d*]oxepin–7(6*H*)–one, (Fig. 1), was prepared from the previously synthesized 2–(3',4',5–trimethoxy biphenyl–2–yloxy) acetyl chloride.

The dihedral angle between the benzene ring (C1–C6) and benzene ring (C7–C12) is 34.55 (5)°. In the crystal structure neither classical nor non–classical hydrogen bonds are found.

### Experimental

To a solution of 2–(3',4',5–trimethoxybiphenyl–2–yloxy) acetyl chloride (5 mmol) in 20 ml trifluoroacetic anhydride was added anhydrous zinc chloride (1 mmol). After stirring the reaction mixture for 12 h at reflux temperature, the trifluoroacetic anhydride was recovered under reduced pressure and residue was added 20 ml water. The aqueous phases were extracted with 100 ml ethyl acetate. The organic extracts were washed with 200 ml saturated aqueous sodium chloride, dried over sodium sulfate, filtered, and concentrated under reduced pressure. The resulting crude material was purified *via* silica gel chromatography (5% ethyl acetate / hexane) to afford a translucent solid in a yield of 80%. Crystals suitable for single–crystal X–ray diffraction were obtained by recrystallization from methanol at room temperature in a total yield of 28%. Analysis found: C 67.9, H 5.3%; C_17_H_16_O_5_ requires: C 67.9, H 5.4%. ^1^H NMR (400 MHz, *DMSO*) 7.33 (s, 1H), 7.25 (d, *J* = 2.9 Hz, 1H), 7.16 (t, *J* = 4.4 Hz, 1H), 6.96 (dd, *J* = 8.7, 2.9 Hz, 1H), 4.75 (s, 2H), 3.97 (s, 1H), 3.86 (s, 1H), 3.82 (s, 2H), 2.50 (s, 2H).

### Refinement

All H atoms were geometrically positioned and refined using a riding model with C—H = 0.93Å, *U*_iso_(H) = 1.2*U*_eq_(C) for aromatic atoms, C—H = 0.97Å, *U*_iso_(H) = 1.2*U*_eq_(C) for CH_2_ atoms, C—H = 0.96Å, *U*_iso_(H) = 1.5*U*_eq_(C) for CH_3_ atoms.

### Crystal data

**Table:**

C_17_H_16_O_5_	*F*(000) = 632
*M**_r_* = 300.30	*D*_x_ = 1.388 Mg m^−^^3^
Monoclinic, *P*2_1_/*c*	Mo *K*α radiation, λ = 0.71073 Å
Hall symbol: -P 2ybc	Cell parameters from 3123 reflections
*a* = 11.5474 (10) Å	θ = 2.7–25.0°
*b* = 8.3776 (7) Å	µ = 0.10 mm^−^^1^
*c* = 14.8801 (13) Å	*T* = 295 K
β = 93.607 (1)°	Block, colorless
*V* = 1436.6 (2) Å^3^	0.32 × 0.26 × 0.24 mm
*Z* = 4	

### Data collection

**Table:**

Bruker SMART APEX II CCD diffractometer	3517 independent reflections
Radiation source: fine–focus sealed tube	2517 reflections with *I* > 2σ(*I*)
graphite	*R*_int_ = 0.022
φ– and ω–scans	θ_max_ = 28.3°, θ_min_ = 2.7°
Absorption correction: multi-scan *SADABS* (Sheldrick, 1996)	*h* = −15→13
*T*_min_ = 0.968, *T*_max_ = 0.976	*k* = −11→11
11231 measured reflections	*l* = −19→18

### Refinement

**Table:**

Refinement on *F*^2^	Primary atom site location: structure-invariant direct methods
Least-squares matrix: full	Secondary atom site location: difference Fourier map
*R*[*F*^2^ > 2σ(*F*^2^)] = 0.044	Hydrogen site location: inferred from neighbouring sites
*wR*(*F*^2^) = 0.127	H-atom parameters constrained
*S* = 1.05	*w* = 1/[σ^2^(*F*_o_^2^) + (0.0578*P*)^2^ + 0.2417*P*] where *P* = (*F*_o_^2^ + 2*F*_c_^2^)/3
3517 reflections	(Δ/σ)_max_ < 0.001
202 parameters	Δρ_max_ = 0.26 e Å^−^^3^
0 restraints	Δρ_min_ = −0.18 e Å^−^^3^

### Special details

**Table:**

Geometry. All s.u.'s (except the s.u. in the dihedral angle between two l.s. planes) are estimated using the full covariance matrix. The cell s.u.'s are taken into account individually in the estimation of s.u.'s in distances, angles and torsion angles; correlations between s.u.'s in cell parameters are only used when they are defined by crystal symmetry. An approximate (isotropic) treatment of cell s.u.'s is used for estimating s.u.'s involving l.s. planes.
Refinement. Refinement of *F*^2^ against ALL reflections. The weighted *R*–factor *wR* and goodness of fit *S* are based on *F*^2^, conventional *R*–factors *R* are based on *F*, with *F* set to zero for negative *F*^2^. The threshold expression of *F*^2^ > σ(*F*^2^) is used only for calculating *R*–factors(gt) *etc*. and is not relevant to the choice of reflections for refinement. *R*–factors based on *F*^2^ are statistically about twice as large as those based on *F*, and *R*–factors based on ALL data will be even larger.

### Fractional atomic coordinates and isotropic or equivalent isotropic displacement parameters (Å^2^)

**Table:**

	*x*	*y*	*z*	*U*_iso_*/*U*_eq_	
C1	0.64848 (13)	0.13216 (17)	0.18464 (10)	0.0430 (3)	
C2	0.53334 (14)	0.09007 (19)	0.18643 (11)	0.0509 (4)	
H2	0.4842	0.1508	0.2200	0.061*	
C3	0.48957 (14)	−0.0412 (2)	0.13909 (11)	0.0515 (4)	
H3	0.4115	−0.0682	0.1398	0.062*	
C4	0.56459 (14)	−0.13171 (19)	0.09053 (11)	0.0477 (4)	
C5	0.67990 (14)	−0.08725 (18)	0.08744 (11)	0.0466 (4)	
H5	0.7286	−0.1475	0.0533	0.056*	
C6	0.72465 (13)	0.04551 (17)	0.13424 (9)	0.0409 (3)	
C7	0.84963 (13)	0.08803 (16)	0.13366 (9)	0.0396 (3)	
C8	0.93022 (13)	−0.03654 (16)	0.12805 (10)	0.0426 (3)	
H8	0.9037	−0.1414	0.1265	0.051*	
C9	1.04778 (13)	−0.00803 (16)	0.12484 (10)	0.0422 (3)	
C10	1.08923 (13)	0.15007 (17)	0.12771 (10)	0.0415 (3)	
C11	1.01109 (13)	0.27300 (16)	0.13109 (10)	0.0428 (3)	
H11	1.0380	0.3776	0.1309	0.051*	
C12	0.89187 (13)	0.24533 (16)	0.13480 (10)	0.0403 (3)	
C13	0.81824 (14)	0.39121 (18)	0.13527 (11)	0.0480 (4)	
C14	0.71248 (14)	0.39826 (18)	0.18854 (11)	0.0520 (4)	
H14A	0.7209	0.4871	0.2302	0.062*	
H14B	0.6457	0.4201	0.1475	0.062*	
C15	0.41610 (16)	−0.3191 (2)	0.04808 (13)	0.0642 (5)	
H15A	0.4005	−0.3388	0.1097	0.096*	
H15B	0.4046	−0.4156	0.0139	0.096*	
H15C	0.3644	−0.2381	0.0237	0.096*	
C16	1.09330 (15)	−0.28365 (17)	0.11482 (13)	0.0558 (4)	
H16A	1.0540	−0.3101	0.1678	0.084*	
H16B	1.1596	−0.3518	0.1108	0.084*	
H16C	1.0414	−0.2983	0.0625	0.084*	
C17	1.25317 (15)	0.32212 (19)	0.12655 (14)	0.0615 (5)	
H17A	1.2195	0.3802	0.0758	0.092*	
H17B	1.3358	0.3170	0.1230	0.092*	
H17C	1.2354	0.3754	0.1811	0.092*	
O1	0.68972 (10)	0.25851 (13)	0.23796 (7)	0.0506 (3)	
O2	0.53211 (10)	−0.26715 (14)	0.04357 (9)	0.0641 (4)	
O3	1.13016 (9)	−0.12153 (12)	0.11976 (9)	0.0558 (3)	
O4	1.20699 (9)	0.16530 (12)	0.12625 (8)	0.0527 (3)	
O5	0.84540 (10)	0.51664 (13)	0.09494 (9)	0.0650 (4)	

### Atomic displacement parameters (Å^2^)

**Table:**

	*U*^11^	*U*^22^	*U*^33^	*U*^12^	*U*^13^	*U*^23^
C1	0.0423 (8)	0.0459 (8)	0.0414 (8)	0.0005 (6)	0.0062 (6)	−0.0007 (6)
C2	0.0459 (9)	0.0560 (9)	0.0521 (9)	0.0024 (7)	0.0131 (7)	−0.0034 (7)
C3	0.0379 (8)	0.0620 (10)	0.0553 (10)	−0.0048 (7)	0.0089 (7)	0.0018 (8)
C4	0.0464 (9)	0.0482 (8)	0.0488 (9)	−0.0073 (7)	0.0056 (7)	−0.0026 (7)
C5	0.0430 (9)	0.0461 (8)	0.0514 (9)	−0.0021 (7)	0.0097 (7)	−0.0063 (7)
C6	0.0408 (8)	0.0408 (7)	0.0416 (8)	−0.0006 (6)	0.0056 (6)	0.0007 (6)
C7	0.0395 (8)	0.0395 (7)	0.0400 (7)	−0.0004 (6)	0.0043 (6)	−0.0003 (6)
C8	0.0433 (8)	0.0332 (7)	0.0515 (9)	−0.0012 (6)	0.0044 (6)	−0.0002 (6)
C9	0.0411 (8)	0.0335 (7)	0.0519 (9)	0.0031 (6)	0.0025 (6)	0.0009 (6)
C10	0.0368 (8)	0.0371 (7)	0.0508 (8)	−0.0004 (6)	0.0038 (6)	0.0027 (6)
C11	0.0431 (9)	0.0325 (7)	0.0530 (9)	−0.0019 (6)	0.0036 (7)	0.0004 (6)
C12	0.0410 (8)	0.0360 (7)	0.0439 (8)	0.0033 (6)	0.0037 (6)	0.0000 (6)
C13	0.0451 (9)	0.0390 (8)	0.0593 (10)	0.0050 (6)	−0.0006 (7)	−0.0025 (7)
C14	0.0510 (10)	0.0454 (8)	0.0598 (10)	0.0061 (7)	0.0050 (8)	−0.0094 (7)
C15	0.0527 (11)	0.0744 (12)	0.0652 (11)	−0.0214 (9)	0.0006 (9)	−0.0076 (9)
C16	0.0530 (10)	0.0328 (8)	0.0812 (12)	0.0027 (7)	0.0023 (9)	−0.0053 (7)
C17	0.0462 (10)	0.0419 (9)	0.0967 (14)	−0.0078 (7)	0.0061 (9)	0.0024 (9)
O1	0.0527 (7)	0.0506 (6)	0.0492 (6)	−0.0018 (5)	0.0093 (5)	−0.0104 (5)
O2	0.0521 (7)	0.0640 (8)	0.0771 (8)	−0.0187 (6)	0.0115 (6)	−0.0191 (6)
O3	0.0422 (6)	0.0329 (5)	0.0922 (9)	0.0045 (4)	0.0037 (6)	−0.0005 (5)
O4	0.0371 (6)	0.0360 (5)	0.0852 (8)	−0.0015 (4)	0.0064 (5)	0.0038 (5)
O5	0.0541 (7)	0.0410 (6)	0.1010 (10)	0.0040 (5)	0.0143 (7)	0.0148 (6)

### Geometric parameters (Å, °)

**Table:**

C1—C2	1.377 (2)	C11—C12	1.401 (2)
C1—O1	1.3889 (18)	C11—H11	0.9300
C1—C6	1.395 (2)	C12—C13	1.4891 (19)
C2—C3	1.385 (2)	C13—O5	1.2595 (19)
C2—H2	0.9300	C13—C14	1.498 (2)
C3—C4	1.388 (2)	C14—O1	1.4159 (19)
C3—H3	0.9300	C14—H14A	0.9700
C4—O2	1.3724 (19)	C14—H14B	0.9700
C4—C5	1.386 (2)	C15—O2	1.414 (2)
C5—C6	1.394 (2)	C15—H15A	0.9600
C5—H5	0.9300	C15—H15B	0.9600
C6—C7	1.487 (2)	C15—H15C	0.9600
C7—C8	1.4042 (19)	C16—O3	1.4237 (17)
C7—C12	1.4049 (19)	C16—H16A	0.9600
C8—C9	1.382 (2)	C16—H16B	0.9600
C8—H8	0.9300	C16—H16C	0.9600
C9—O3	1.3504 (17)	C17—O4	1.4177 (18)
C9—C10	1.4081 (19)	C17—H17A	0.9600
C10—O4	1.3674 (18)	C17—H17B	0.9600
C10—C11	1.372 (2)	C17—H17C	0.9600
			
C2—C1—O1	118.73 (13)	C11—C12—C13	115.30 (12)
C2—C1—C6	121.28 (14)	C7—C12—C13	124.89 (14)
O1—C1—C6	119.90 (14)	O5—C13—C12	121.55 (14)
C1—C2—C3	121.03 (15)	O5—C13—C14	117.05 (13)
C1—C2—H2	119.5	C12—C13—C14	121.30 (14)
C3—C2—H2	119.5	O1—C14—C13	115.23 (13)
C2—C3—C4	118.62 (15)	O1—C14—H14A	108.5
C2—C3—H3	120.7	C13—C14—H14A	108.5
C4—C3—H3	120.7	O1—C14—H14B	108.5
O2—C4—C5	115.95 (14)	C13—C14—H14B	108.5
O2—C4—C3	123.86 (15)	H14A—C14—H14B	107.5
C5—C4—C3	120.19 (14)	O2—C15—H15A	109.5
C4—C5—C6	121.63 (14)	O2—C15—H15B	109.5
C4—C5—H5	119.2	H15A—C15—H15B	109.5
C6—C5—H5	119.2	O2—C15—H15C	109.5
C5—C6—C1	117.21 (14)	H15A—C15—H15C	109.5
C5—C6—C7	121.17 (13)	H15B—C15—H15C	109.5
C1—C6—C7	121.56 (13)	O3—C16—H16A	109.5
C8—C7—C12	117.84 (13)	O3—C16—H16B	109.5
C8—C7—C6	117.99 (13)	H16A—C16—H16B	109.5
C12—C7—C6	124.12 (13)	O3—C16—H16C	109.5
C9—C8—C7	121.98 (13)	H16A—C16—H16C	109.5
C9—C8—H8	119.0	H16B—C16—H16C	109.5
C7—C8—H8	119.0	O4—C17—H17A	109.5
O3—C9—C8	125.25 (13)	O4—C17—H17B	109.5
O3—C9—C10	115.14 (13)	H17A—C17—H17B	109.5
C8—C9—C10	119.62 (13)	O4—C17—H17C	109.5
O4—C10—C11	125.97 (13)	H17A—C17—H17C	109.5
O4—C10—C9	115.07 (12)	H17B—C17—H17C	109.5
C11—C10—C9	118.95 (13)	C1—O1—C14	113.68 (12)
C10—C11—C12	121.83 (13)	C4—O2—C15	117.35 (14)
C10—C11—H11	119.1	C9—O3—C16	117.73 (12)
C12—C11—H11	119.1	C10—O4—C17	117.43 (12)
C11—C12—C7	119.74 (13)		
